# Analysis of 19 Highly Conserved *Vibrio cholerae* Bacteriophages Isolated from Environmental and Patient Sources Over a Twelve-Year Period

**DOI:** 10.3390/v10060299

**Published:** 2018-06-01

**Authors:** Angus Angermeyer, Moon Moon Das, Durg Vijai Singh, Kimberley D. Seed

**Affiliations:** 1 Department of Plant and Microbial Biology, University of California Berkeley, Berkeley, CA 94720, USA; angermeyer@berkeley.edu; 2 Department of Infectious Disease Biology, Institute of Life Sciences, Nalco Square, Bhubaneswar 751023, India; moon.scial@gmail.com (M.M.D.); durg.singh@gmail.com (D.V.S.); 3 Chan Zuckerberg Biohub, San Francisco, CA 94158, USA

**Keywords:** cholera, bacteriophage, *Vibrio cholerae*, pan-genome, myovirus, phylogenetics, vibrio phage

## Abstract

The *Vibrio cholerae* biotype “El Tor” is responsible for all of the current epidemic and endemic cholera outbreaks worldwide. These outbreaks are clonal, and it is hypothesized that they originate from the coastal areas near the Bay of Bengal, where the lytic bacteriophage ICP1 (International Centre for Diarrhoeal Disease Research, Bangladesh cholera phage 1) specifically preys upon these pathogenic outbreak strains. ICP1 has also been the dominant bacteriophage found in cholera patient stools since 2001. However, little is known about the genomic differences between the ICP1 strains that have been collected over time. Here, we elucidate the pan-genome and the phylogeny of the ICP1 strains by aligning, annotating, and analyzing the genomes of 19 distinct isolates that were collected between 2001 and 2012. Our results reveal that the ICP1 isolates are highly conserved and possess a large core-genome as well as a smaller, somewhat flexible accessory-genome. Despite its overall conservation, ICP1 strains have managed to acquire a number of unknown genes, as well as a CRISPR-Cas system which is known to be critical for its ongoing struggle for co-evolutionary dominance over its host. This study describes a foundation on which to construct future molecular and bioinformatic studies of these *V. cholerae*-associated bacteriophages.

## 1. Introduction

*Vibrio cholerae* is a globally-distributed bacterium and is the causal agent of the disease cholera, which is a potentially severe intestinal illness that affects ~1–5 million people and is responsible for up to ~140,000 deaths annually [1]. The ongoing (seventh) pandemic is comprised of the *V. cholerae* biotype “El Tor” (predominantly serotype O1) and is responsible for current endemic and epidemic disease [2]. Epidemic disease outbreaks sweep across the globe periodically. This has been traced back to a single lineage that has emerged from the Bay of Bengal region in multiple waves over the last half-century [3]. Despite the overall genetic heterogeneity of this lineage in which individual outbreaks are nearly always clonal [4], there is an abundance of subtle variation and horizontal transfer that has been observed between outbreaks over time [5,6]. It is hypothesized that the Bay of Bengal serves as a reservoir where the El Tor strains circulate throughout the year, and exchange genetic material and undergo ecological selection before infiltrating coastal communities. They are subsequently transported by infected individuals to larger cities where they can be transmitted globally [5,7]. This mechanism is thought to create a bottleneck for strains, and results in the clonality of outbreaks.

Bacteriophage, viruses that uniquely infect bacteria, are extremely abundant in the environment and can outnumber their prokaryotic hosts by several orders of magnitude [8]. As such, bacteriophage plays a key role in the evolution of their hosts through both selection and phage-mediated lateral gene transfer [9]. These processes are likely to be very important to *V. cholerae* strain evolution in the Bay of Bengal as well. Previous work has identified a *V. cholerae* O1-specific [10] lytic *Myoviridae* bacteriophage called ICP1 (International Centre for Diarrhoeal Disease Research, Bangladesh cholera phage 1) to be of particular interest in this system [11]. In Bangladesh, ICP1 has been found in water samples [12,13] and has been identified as the dominant phage in cholera patient stool samples since 2001 [11]. The persistence of this phage over time indicates that *V. cholerae* has strategies to limit ICP1 predation, and that ICP1 can evolve to overcome such defenses. Indeed, from this natural genetic laboratory, several complex and surprising adaptations/acquisitions have occurred in the race for survival between *V. cholerae* and ICP1. These include self-mobilizing chromosomal islands that can provide a rapid and efficient response to ICP1 infection [14], as well as the first known example of a bacteriophage-encoded CRISPR-Cas system [15]. Initial characterization of eight ICP1 isolates that were collected between 2001–2011 noted the relatively low level of diversity and lack of major genomic rearrangements, deletions, or insertions [11] (with the exception of its remarkable CRISPR-Cas acquisition [15]). Here, we build upon the initial characterization of ICP1 to perform a comparative genomic analysis on 19 individual ICP1 isolates to reconstruct their phylogenetic relationships over time, to identify the core and accessory genomes, and to infer possible gene function where possible.

*V. cholerae* is an organism that affects millions and appropriately, it is well-studied with modern bioinformatic and sequencing tools. It is important that their concomitant bacteriophages are similarly studied to help us better elucidate the important role that bacteriophage likely plays in the evolution of *V. cholerae,* and to explain the epidemiology of the ongoing cholera pandemic.

## 2. Materials and Methods

Nineteen ICP1 bacteriophage genomes were acquired from various sources (Table 1). Those genomes that are not specifically described below were downloaded from the NCBI GenBank database. Their metadata (if available) was used to inform our reporting of isolation source and isolation year. In cases where this information differed from what was reported in a genome’s original publication, we deferred to the GenBank database. The Isolation year was used to standardize the ICP1 bacteriophage naming convention: ICP1_YEAR_X where “X” was sequentially assigned letter (A–Z) based on the order in which the genomes were named. The only exception was the original “ancestral” ICP1 isolate, which was simply referred to as “ICP1” [11].

Genomes that were not already available on GenBank included: ICP1_2006_E and ICP1_2011_A, which were isolated and sequenced as described previously [15]. ICP1_2011_A was assembled using CLC Genomics Workbench v10 (Qiagen, Redwood City, CA, USA), and ICP1_2006_E was assembled with IDBA-UD v1.1.3 [16] (default settings) after fastq read filtering with USEARCH v10.0.240 [17] fastq_filter (-fastq_maxee_rate 0.001 -fastq_maxns 1 -fastq_truncqual 15 -fastq_maxee 0.25). ICP1_2011_B was assembled from an existing diarrheal stool metagenomic sample [18] (SRA: PRJEB9150; Run: ERR866578) using USEARCH filtering and IDBA-UD de novo assembly as above (same parameters), to generate an incomplete ICP1 contig. That contig was then used as a reference sequence to reassemble the same filtered reads using IDBA-Hybrid [16] with default settings. Finally, ICP1_2012_A was isolated from a cholera patient stool sample that was collected in Silvassa, India [19]. Genomic DNA libraries were sequenced using an Illumina Mi-Seq (Genotypic Technology, Bangalore, India). Genome assembly was performed with CLC Genomics Workbench v10.

Whole-genome alignment was performed on all 19 genomes using progressiveMauve [20] (build: 25 February 2015) with default settings. The Mauve xmfa alignment output file was first converted to a Phylip format using BioPython v1.71 [21]. A maximum likelihood phylogenetic tree (unrooted) was then constructed and supported by bootstrap analysis (-s BEST --rand_start --n_rand_starts 10 -b 100) using PhyML v20120412 [22] with the default substitution model (HKY85). A companion phylogeny based on the concatenated core-genome (described below) was also constructed using the same methods. Dendroscope3 v3.5.9 [23] was used to generate a tanglegram that joined both the whole-genome and core-genome trees with ICP1 ancestral manually set as the outgroup. The whole-genome alignment was also used to determine a consensus ICP1 sequence using CLC Genomics Workbench v10, which all ICP1 genomes were visually mapped back onto using BRIG v0.95 [24].

All extant CDS annotations, hereafter referred to as “Open Reading Frames” (ORFs), in the ICP1 ancestral GenBank file were blasted (BLASTn v2.6.0 [25]) against every other ICP1 genomic sequence to find homologous ORFs. Putative hits were considered homologs, and were annotated as such if the following conditions were met: the subject hit was a complete ORF (start and stop codons; codon table = 11), *E*-value ≤ 1 × 10^−10^, % identity ≥85, and the two matched ORFs were within a 10% sequence length of each other. After identifying existing homologs, de novo ORF prediction was performed on all genomes to find additional possible coding sequences using Prodigal v2.6.3 [26] with default settings and a confidence cutoff ≥95%. All of the newly identified putative-ORFs from each genome were then blasted against each other with the same conditions as above, were grouped by homology, and were given an iterative numerical identifier based on their locations in the genome relative to the extant ORFs (e.g., ORF1, ORF2, ORF2.1, ORF2.2, ORF3, etc.). ORFs were then categorized into groups based on how many of the 19 genomes they were found in. If an ORF was present in all 19 genomes, it was considered to be part of the core-genome. Otherwise, it was considered to be part of the accessory-genome. Core and accessory ORFs were also mapped to the BRIG alignment. The core-genome ORF protein sequences were concatenated by strain to create a core-genomic, pseudo-genome for each ICP1 strain and were subjected to the phylogenetic analysis, as above. The core and accessory ORFs were also queried against the NCBI’s Conserved Domain Database (https://www.ncbi.nlm.nih.gov/cdd) to determine whether any of them possessed interesting domain homology that was not detected by BLAST alone.

## 3. Results

### 3.1. Genome Characteristics and Phylogeny

**Figure 1:**
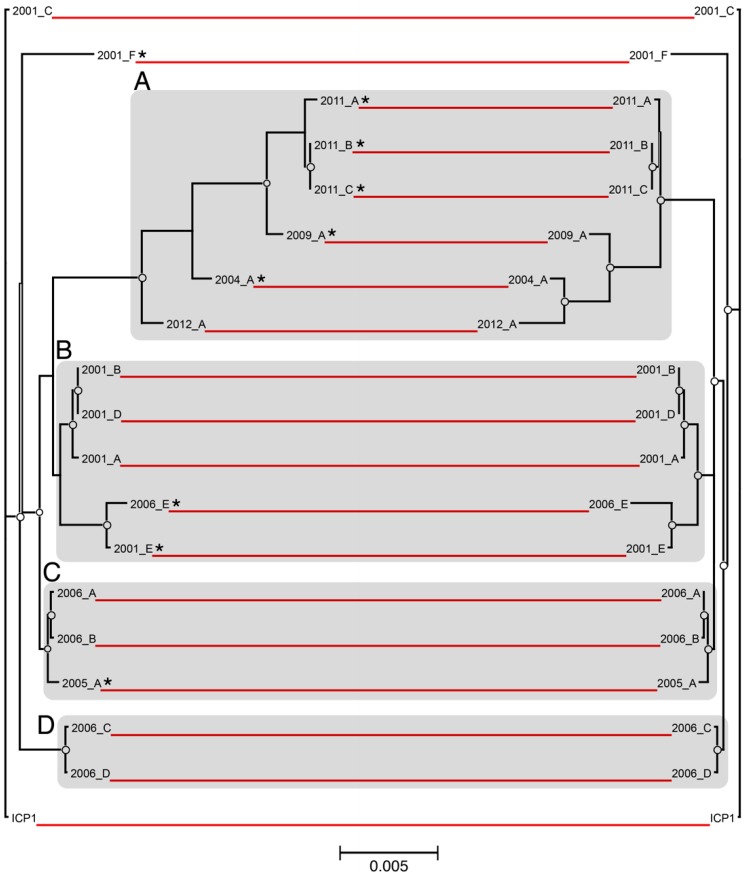
Phylogenetic comparison of ICP1 whole and core-genomes. Maximum likelihood phylogenetic trees (unrooted) were constructed based on a multiple whole-genome alignment of all 19 ICP1 phage sequences (**left**), as well as an alignment of the concatenated ORFs for each phage that comprise the core genome (**right**). The red lines connect identical leaves between trees to indicate relative phylogeny. The circles represent nodes with ≥90% bootstrap support (*n* = 100). The scale bar measures nucleotide substitutions per base pair. Distinct phylogenetic clusters are shaded grey, and CRISPR strains are marked with “*”.

ICP1 genomes were analyzed from isolates that were collected over a 12-year period, from 2001 to 2012 (Table 1). The isolates were derived from both environmental water samples and patient stool samples that were collected in Bangladesh (*n* = 18) and India (ICP1_2012_A). Genome length was slightly variable with an average of 126,384 bp (stdev: 3008 bp). The maximum likelihood phylogenetic analysis grouped both of the whole-genome and core-genome alignments into several general clusters (Figure 1). Cluster A contains the five most recently isolated strains, as well as ICP1_2004_A. This cluster also contains five of the seven total CRISPR-positive strains in the dataset. Cluster B contains four of the six 2001 isolates, ICP1_2006_E, and two of the CRISPR-positive strains. Clusters C and D contain isolates from the intermediate years of 2005 and 2006, and one CRISPR-positive is represented in cluster C. The CRISPR-positive strain ICP1_2001_F did not cluster with other isolates. ICP1 ancestral and ICP1_2001C also cluster closely together, however they are not specifically highlighted.

Overall, the topologies between the whole-genome and the core-genome trees were highly similar, with only minor exceptions. Both shared identical clustering and had almost no leaf-level differences within clusters B, C, and D. Cluster A showed an inversion of the phylogenetic differences of the leaves between the two alignments and an increased level of intra-group divergence when the core and accessory-genomes were included (Figure 1, left side). For instance, ICP1_2012_A clustered more closely with other members of group A when only core genomic divergence was considered. Yet, when the commonality of the accessory gene complement was also examined, ICP1_2012_A was the least strongly clustered.

### 3.2. Genome Alignment Visualization

**Figure 2:**
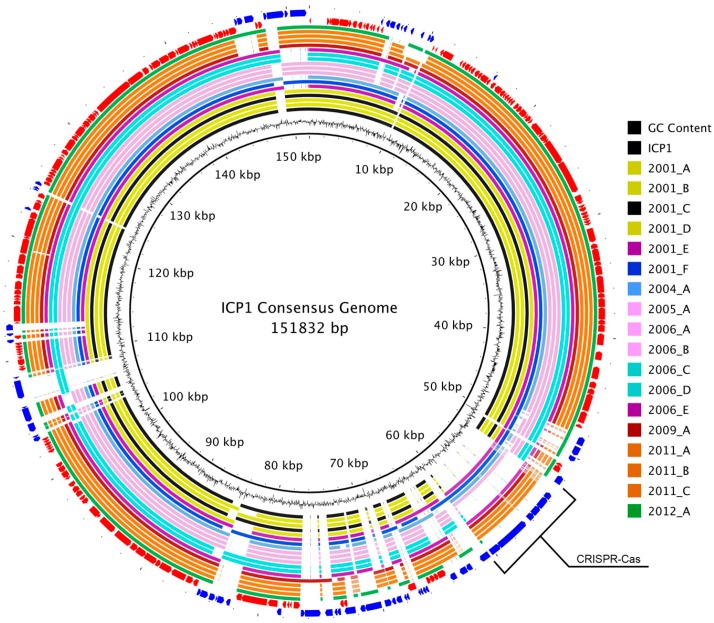
ICP1 pan-genome consensus alignment. A BLASTn-based whole genome alignment of all 19 ICP1 phage genomes using the MAUVE alignment consensus sequence as a reference. The innermost ring is the consensus sequence. The next ring represents the GC content for that region. The following 19 rings display the alignment for each genome and are colored by phylogenetic similarity as determined by the analysis of whole genomes (Figure 1, left). The second to last ring (red) represents the core-genomic ORFs, while the outermost ring (blue) is the accessory-genome ORFs. The CRISPR-Cas insertion region is labeled.

The consensus genomic sequence that was constructed from the whole-genome alignment was 151,832 bp long and contained all of the coding and non-coding regions from each genome. It was used as a reference to map the genomes and to visualize the overall multiple-genome alignment (Figure 2). Variable regions of insertions and deletions were visible as gaps in the circular alignment. Similar to the whole-genome phylogeny, there was not a clear progression of sequence divergence based on isolation chronology. No large regions of GC content difference were observed.

### 3.3. ORF Annotation

Among all 19 genomes, a total of 269 distinct ORFs (based on homology cutoffs) were identified. Of these, 230 were originally annotated in the ICP1 ancestral genome and 39 were called by Prodigal. The number of ORFs per genome varied from 215 to 232 and demonstrated a slight but significant inverse relationship with the year of isolation, i.e., more recent strains had fewer ORFs (Figure S1A). A weaker significant positive trend was observed between number of ORFs and genome length (Figure S1B). This is likely due to the CRISPR-Cas insertions, however there was not a statistically significant correlation between genome length and isolation year.

### 3.4. Core and Accessory Genome Analysis

**Figure 3:**
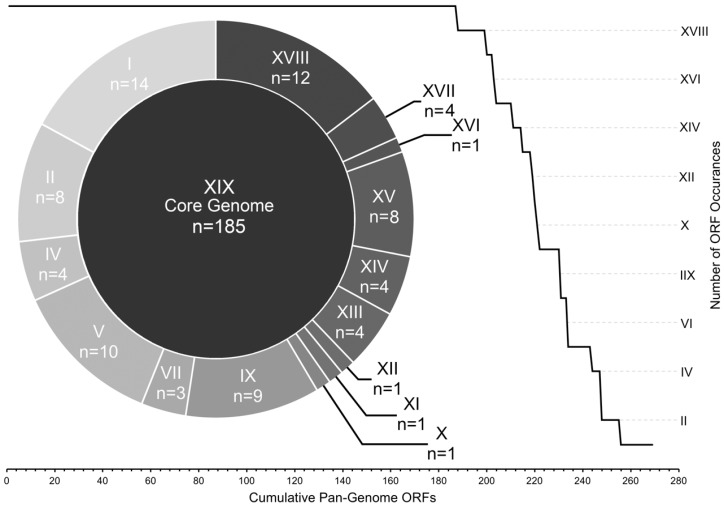
ICP1 pan-genome ORF allocation. All ICP1 ORFs are arranged by the number of genomes in which they were detected. ORF bins are represented by roman numerals, i.e., ‘XIX’ ORFs were found in all 19 genomes (core), “X” ORFs were found in exactly 10 genomes, etc. The line graph demonstrates the overall cumulative curve of core and accessory ORF prevalence. The doughnut chart provides the exact number of ORFs within each accessory bin.

To estimate gene diversity among the genomes, we divided the total pan-genomic ORF complement into core and accessory groups. The core ORFs—those that were found in all 19 genomes—comprised ~70% of the total number of ORFs (185 out of 269), while the other 84 ORFs were considered to be accessory (Figure 3).

**Figure 4:**
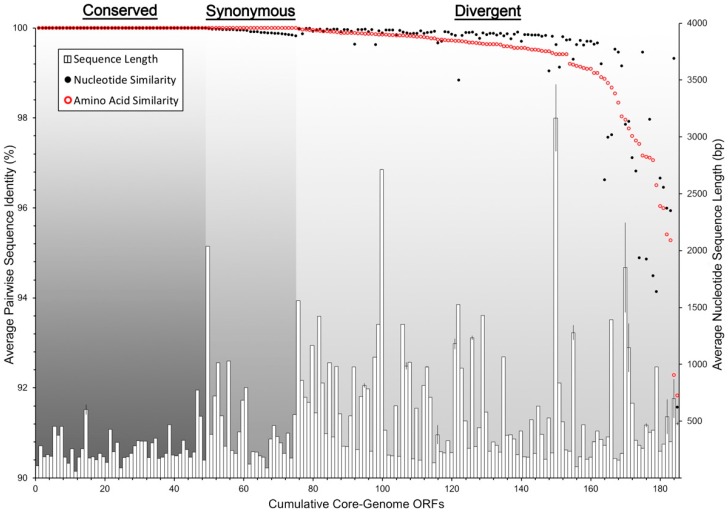
ICP1 core-genome ORF divergence. All ICP1 core-genome ORFs are arranged by the average pairwise similarity (171 pairwise comparisons) of both DNA nucleotide sequence alignments (black dots) and amino acid residue alignments (red circles). The histogram shows the average nucleotide sequence length and the standard deviation among the 19 sequences per ORF. The graph is divided into three sections by types of ORF similarity: Conserved (identical nucleotide and amino acid sequences), Synonymous (identical amino acid sequences, but silent nucleotide mutations), and Divergent (dissimilarity in both nucleotide and amino acid sequences).

Despite their occurrence across all genomes in this study, the core-genome ORFs were not all equally conserved at the sequence level. By comparing average pairwise nucleotide and amino acid sequence identity, we were able to resolve the core ORFs into three distinct groups: conserved-core, synonymous-core, and divergent-core (Figure 4). The conserved-core was comprised of 49 ORFs that shared perfect sequence identity among all of the genomic sequences. Correspondingly, they shared identical amino acid sequence identity. The synonymous-core included 26 ORFs that possessed a small amount of nucleotide diversity between genomes, however all mutations were silent, and the amino acid primary structure was therefore identical. Finally, the divergent-core contained the remaining 110 ORFs that were diverse in both nucleotide and amino acid pairwise identity. These divergent ORFs encompassed a range of pairwise identity similarities from 99.97% to 91.57% at the nucleotide level, and 99.98% to 91.83% for amino acid sequences (Table S1). The degree of pairwise identity difference was not correlated with an ORF’s sequence length (Figure 4).

The accessory ORFs were distributed among all 19 genomes in a complex manner (Table S2) and were grouped into 15 levels of occurrence. These ranged from occurrences in 18 genomes (*n* = 12) to singletons (*n* = 14) (Figure 3), with several patterns of accessory ORF co-occurrence (Table S2). The most obvious example was the CRISPR-Cas locus which was previously known to reside in eight of these genomes [13,15] and was confirmed through our annotation methods. Other examples of sequential ORF co-occurrence included a locus containing ORFs 115, 116, 117, 117.1, and 118 which were found in the same five genomes, ORFs 160, 162, 163 which were found in a different set of five overlapping genomes, and ORFs 222, 223, 225 which were found in 15 genomes.

### 3.5. Conserved Functional Domains

The majority of ORFs in both the core and accessory genomes were classified as hypothetical due to a lack of an informative BLAST identification. Only 18 of the core ORFs (9.7%) currently had a predicted function, with two in the conserved-core, three in the synonymous-core, and 13 in the divergent-core (Table S1). The accessory core contains 11 ORFs with a putative or known function (Table S2). The NCBI conserved domain search identified 18 additional core-genome ORFs and 11 accessory-genome ORFs that shared at least partial amino acid sequence homology to known functional domains.

## 4. Discussion

ICP1 is a *V. cholerae*-infecting bacteriophage that appears to be prevalent throughout the Bay of Bengal’s coastal areas and is transferred readily alongside *V. cholerae* into humans during cholera outbreaks in the region [11]. It is becoming increasingly theorized that this region is the source of most, if not all, global cholera outbreaks [3,4,27], and therefore gaining a better understanding of this co-evolving predator is an important area of ongoing cholera research. In this study, we have performed a comprehensive phylogenetic analysis on all available, well-sequenced ICP1 isolates to elucidate their genetic divergence over time, and to provide a platform on which to develop future ICP1-related bioinformatic analyses.

We have found that the genomes of ICP1 were surprisingly well-conserved between all isolates over the twelve-year period in which they were isolated. This is demonstrated in the whole-genome phylogeny which, while resolvable into distinct phylogenetic clusters, still only represents a maximum variation of approximately one nucleotide substitution per 100 base pairs between the most divergent isolates (Figure 1)—many of which are likely silent or non-coding. A high degree of genomic conservation is also indicated by the relatively large core-genome that is shared between all isolates (Figure 3). This conservation is not only surprising due to the amount of time that the core-genome remained stable but is also surprising due to the complex conditions that likely exist in the coastal and ocean environments where ICP1 is competing against a host population that is almost certainly more diverse than clonal outbreak strains. In at least one other study that examined multiple strains of a single marine bacteriophage (Far-T4) it was shown that sequence variability among strains was at least an order of magnitude greater than we observed with ICP1 [28]. In contrast, a study from a less variable environment found that strains of a *Y. enterocolitica*-infecting podovirus were more highly conserved than ICP1 [29]. However, it must be considered that these environmental pressures may actually be what drives this bacteriophage to maintain a large core-genome, of which the vast majority of ORFs are hypothetical. This large gene complement may be providing the flexibility needed to compete in the Bay of Bengal. Currently, we can only speculate about the exact reason for the conservation of ICP1’s genome, however expanding the repertoire of well-sequenced genomes will help to put a finer constraint on the core-genome and will perhaps identify genetic targets for future study.

Despite the stable conservation of the core-genome, ICP1 also possesses a diverse collection of accessory genes that have been integrated into several locations around the pan-genome (Figure 2). These accessory ORFs appear to be both single acquisitions, as well as integrations of larger loci (Table S2). The most notable of the latter is the previously described acquisition of a CRIPSR-Cas system which is used as a weapon in the arms race between ICP1 and *V. cholerae* [14,15]. This may suggest that other mechanisms of molecular warfare would be likely targets for acquisition, however if so, our conserved domain analyses failed to reveal mechanisms that were already known. Interestingly though, the trend has been for genomic ORF counts to diminish over time, culminating in the latest isolate from India in 2012, which possessed the fewest overall number of ORFs, i.e., the smallest accessory-genome (Figure S1). This could be due to the shedding of outdated or detrimental genes, possibly because the acquisition of a CRISPR-Cas system provided enough flexibility to obviate the need for other mechanisms. Furthermore, although ICP1_2012_A is CRISPR-Cas negative, the other five most recent isolates are positive. However, it is also possible that this is a regional difference, and that more sequenced genomes will help to determine whether this trend is chronological, geographical, or spurious.

Querying the ORFs against the NCBIs conserved domain database returned several hits to do with replication, nucleotide metabolism, recombination, endonuclease activity, and a few other basic functions (Tables S1 and S2). However, what may be the most telling is that 80% of all ORFs do not possess homology to any conserved domain, indicating that there is a great deal left to be learnt about the interaction between ICP1 and *V. cholerae*. It should also be noted that the annotation of ORFs necessarily requires certain assumptions to be made about similarity cutoffs and thresholds and, as such, these ORF calls are best viewed as estimates. However, we were conservative in our methods and are confident that a very large proportion of the calls are accurate.

As we advance our understanding of how cholera spreads globally, it will also be important to continue to track ICP1’s phylogeny and genetic composition. This will help us to develop a better understanding of ICP1’s co-evolution with *V. cholerae* and will assist us in disentangling the complex molecular and ecological interactions that may play an important role in defining cholera outbreaks.

## Figures and Tables

**Table 1:** ICP1 (International Centre for Diarrhoeal Disease Research, Bangladesh cholera phage 1) bacteriophage strains.

Standardized Name	Previous Name	Isolation Year	Isolation Source	Genome Size (bp)	GenBank Accession	Genome Citation
ICP1	-	2001	Stool	125,956	HQ641347	Seed et al., 2011
ICP1_2001_A	-	2001	Stool	124,826	HQ641353	Seed et al., 2011
ICP1_2001_B	JSF1	2001	Water	126,082	KY883636	Naser et al., 2017
ICP1_2001_C	JSF2	2001	Water	126,082	KY883637	Naser et al., 2017
ICP1_2001_D	JSF4	2001 *	Water *	124,261	KY065147	Naser et al., 2017
ICP1_2001_E	JSF5	2001 *	Water	132,142	KY883634	Naser et al., 2017
ICP1_2001_F	JSF6	2001 *	Water	133,685	KY883635	Naser et al., 2017
ICP1_2004_A	-	2004	Stool	128,083	HQ641354	Seed et al., 2011
ICP1_2005_A	-	2005	Stool	129,373	HQ641352	Seed et al., 2011
ICP1_2006_A	-	2006	Stool	123,104	HQ641351	Seed et al., 2011
ICP1_2006_B	-	2006	Stool	123,097	HQ641350	Seed et al., 2011
ICP1_2006_C	-	2006	Stool	124,497	HQ641349	Seed et al., 2011
ICP1_2006_D	-	2006	Stool	124,497	HQ641348	Seed et al., 2011
ICP1_2006_E	-	2006	Stool	128,298	MH310934	This study
ICP1_2009_A	JSF13	2009	Water	128,814	KY883638	Naser et al., 2017
ICP1_2011_A	-	2011	Stool	126,861	MH310933	This study
ICP1_2011_B	-	2011	Stool	125,128	MH310935	This study
ICP1_2011_C	JSF14	2011	Water	125,096	KY883639	Naser et al., 2017
ICP1_2012_A	-	2012	Stool	121,418	MH310936	This study

* GenBank metadata were reported in cases where they differed from the original publication.

## Supplementary Materials

The following are available online at http://www.mdpi.com/1999-4915/10/6/299/s1, Figure S1: ORF occurrence trends by year and genome length, Table S1: Core-genome ORFs pairwise similarity and CDD hits, Table S2: Accessory-genome ORFs ICP1 strain matrix.
