# Supplementary material for: Analysis of 19 Highly Conserved Vibrio cholerae Bacteriophages Isolated from Environmental and Patient Sources Over a Twelve-Year Period
Source: Viruses. 2018 Jun 1;10(6):299. doi: 10.3390/v10060299 (PMC6024749; doi:10.3390/v10060299)
Supplement: Supplementary file 1 [file viruses-10-00299-s001.pdf]

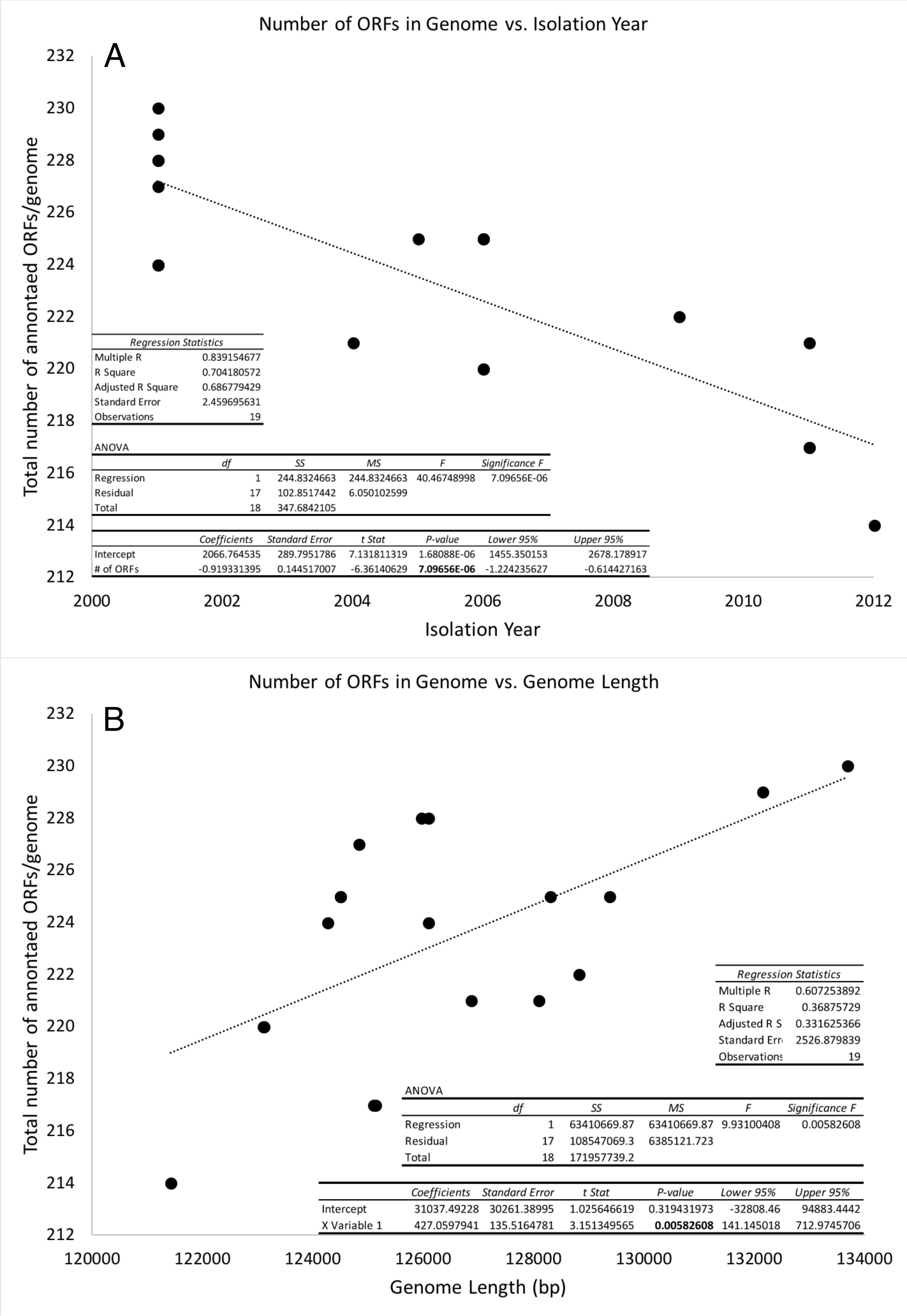

Figure S1: ORF linear regression statistics. ANOVA analyses of the linear regressions between (A) number of ORFs/genome vs genome isolation year and (B) ORFs/genome vs. genome length.

Table S1: Core-genome Information

Group	ORF Name	Nucleotide Similarity	Amino Acid Similarity	nuc_stdev	aa_stdev	Known Function	CDD Domain name	E-Value	PSSM-ID
Conserved Core	ORF1	100.00	100.00	0.00	0.00	-	-	-	-
	ORF10	100.00	100.00	0.00	0.00	-	-	-	-
	ORF109	100.00	100.00	0.00	0.00	-	-	-	-
	ORF12	100.00	100.00	0.00	0.00	-	-	-	-
	ORF13	100.00	100.00	0.00	0.00	-	-	-	-
	ORF134	100.00	100.00	0.00	0.00	-	-	-	-
	ORF135	100.00	100.00	0.00	0.00	-	-	-	-
	ORF138	100.00	100.00	0.00	0.00	-	-	-	-
	ORF142	100.00	100.00	0.00	0.00	-	-	-	-
	ORF154	100.00	100.00	0.00	0.00	-	-	-	-
	ORF158	100.00	100.00	0.00	0.00	-	-	-	-
	ORF164	100.00	100.00	0.00	0.00	-	-	-	-
	ORF167	100.00	100.00	0.00	0.00	-	-	-	-
	ORF168	100.00	100.00	0.00	0.00	-	-	-	-
	ORF171	100.00	100.00	0.00	0.00	-	HAD_like superfamily	1.87E-04	328728
	ORF175	100.00	100.00	0.00	0.00	-	-	-	-
	ORF180	100.00	100.00	0.00	0.00	-	-	-	-
	ORF195	100.00	100.00	0.00	0.00	-	-	-	-
	ORF206	100.00	100.00	0.00	0.00	-	-	-	-
	ORF214	100.00	100.00	0.00	0.00	-	-	-	-
	ORF216	100.00	100.00	0.00	0.00	-	-	-	-
	ORF218	100.00	100.00	0.00	0.00	-	-	-	-
	ORF226	100.00	100.00	0.00	0.00	-	-	-	-
	ORF227	100.00	100.00	0.00	0.00	-	-	-	-
	ORF26	100.00	100.00	0.00	0.00	-	-	-	-
	ORF29	100.00	100.00	0.00	0.00	-	-	-	-
	ORF3	100.00	100.00	0.00	0.00	-	-	-	-
	ORF32	100.00	100.00	0.00	0.00	-	-	-	-
	ORF33	100.00	100.00	0.00	0.00	-	-	-	-
	ORF34	100.00	100.00	0.00	0.00	-	-	-	-
	ORF39	100.00	100.00	0.00	0.00	-	DUF3696 superfamily	6.88E-03	331172
	ORF41	100.00	100.00	0.00	0.00	-	-	-	-
	ORF42	100.00	100.00	0.00	0.00	-	-	-	-
	ORF47	100.00	100.00	0.00	0.00	-	-	-	-
	ORF48	100.00	100.00	0.00	0.00	-	-	-	-
	ORF50	100.00	100.00	0.00	0.00	-	DUF1778 superfamily	5.16E-06	321696
	ORF52	100.00	100.00	0.00	0.00	-	-	-	-
	ORF54	100.00	100.00	0.00	0.00	-	-	-	-
	ORF55	100.00	100.00	0.00	0.00	ribonuclease H	RNase_HI_prokaryote_like	1.19E-56	260010
	ORF56	100.00	100.00	0.00	0.00	-	RNase_H_like superfamily	2.12E-14	326352
	ORF60	100.00	100.00	0.00	0.00	-	-	-	-
	ORF61	100.00	100.00	0.00	0.00	-	-	-	-
	ORF62	100.00	100.00	0.00	0.00	-	-	-	-
	ORF64	100.00	100.00	0.00	0.00	-	-	-	-
	ORF65	100.00	100.00	0.00	0.00	-	-	-	-
	ORF66	100.00	100.00	0.00	0.00	-	Peptidases_S8_S53 superfamily	5.78E-03	324584
	ORF75	100.00	100.00	0.00	0.00	putative baseplate assembly protein	Phage_base_V superfamily	9.89E-03	327437
	ORF82	100.00	100.00	0.00	0.00	-	-	-	-
	ORF9	100.00	100.00	0.00	0.00	-	-	-	-
Synonymous Core	ORF57	99.98	100.00	0.02	0.00	putative primase/helicase	RecA-like_NTPases superfamily	7.80E-28	333705
	ORF166	99.97	100.00	0.08	0.00	-	-	-	-
	ORF80	99.97	100.00	0.06	0.00	HNH homing endonuclease	HNHc superfamily	7.94E-05	320750
	ORF189	99.97	100.00	0.05	0.00	-	DnaQ_like_exo superfamily	1.39E-05	324557
	ORF137	99.96	100.00	0.07	0.00	-	-	-	-
	ORF213	99.96	100.00	0.11	0.00	-	-	-	-
	ORF81	99.96	100.00	0.05	0.00	-	Macoilin superfamily	3.48E-04	313022
	ORF43	99.96	100.00	0.13	0.00	-	-	-	-
	ORF45	99.95	100.00	0.14	0.00	-	-	-	-
	ORF74	99.95	100.00	0.10	0.00	-	-	-	-
	ORF4	99.95	100.00	0.07	0.00	-	-	-	-
	ORF112	99.94	100.00	0.06	0.00	putative DNA-binding protein Roi	Phage_pRha superfamily	2.26E-08	324635
	ORF191	99.91	100.00	0.25	0.00	-	-	-	-
	ORF46	99.91	100.00	0.17	0.00	-	-	-	-
	ORF30	99.91	100.00	0.18	0.00	-	-	-	-
	ORF44	99.90	100.00	0.20	0.00	-	-	-	-
	ORF210	99.89	100.00	0.22	0.00	-	-	-	-
	ORF153	99.88	100.00	0.34	0.00	-	-	-	-
	ORF139	99.88	100.00	0.14	0.00	-	-	-	-
	ORF83	99.88	100.00	0.12	0.00	-	-	-	-
	ORF7	99.88	100.00	0.14	0.00	-	-	-	-
	ORF194	99.87	100.00	0.16	0.00	-	-	-	-
	ORF2	99.86	100.00	0.22	0.00	-	-	-	-
	ORF181	99.85	100.00	0.22	0.00	-	-	-	-
	ORF15	99.84	100.00	0.25	0.00	-	-	-	-
	ORF126	99.82	100.00	0.36	0.00	-	-	-	-
	ORF176	99.97	99.98	0.03	0.06	putative exodeoxyribonuclease	-	-	-
	ORF173	99.87	99.96	0.09	0.11	recombination-associated protein RdgC	RdgC superfamily	5.57E-21	321354
	ORF170	99.99	99.96	0.04	0.13	-	-	-	-
	ORF179	99.98	99.95	0.05	0.14	-	-	-	-
	ORF68	99.92	99.95	0.11	0.11	-	-	-	-
	ORF49	99.96	99.94	0.07	0.16	-	-	-	-
	ORF73	99.96	99.94	0.04	0.10	putative baseplate component	Baseplate_J superfamily	1.29E-05	321435
	ORF196	99.91	99.92	0.12	0.22	putative adenine methyltransferase	Dam	2.70E-32	223415
	ORF131	99.97	99.92	0.08	0.24	-	-	-	-
	ORF76	99.95	99.92	0.07	0.13	-	-	-	-
	ORF71	99.97	99.91	0.08	0.26	-	-	-	-
	ORF211	99.97	99.91	0.05	0.15	ClpP ATP-dependent protease subunit	crotonase-like superfamily	6.26E-19	329030
	ORF120	99.91	99.89	0.10	0.22	-	-	-	-
	ORF63	99.96	99.89	0.11	0.33	-	-	-	-
	ORF132	99.96	99.88	0.11	0.34	-	-	-	-
	ORF177	99.92	99.88	0.13	0.23	-	-	-	-
	ORF79	99.63	99.88	0.55	0.16	-	DUF2130 superfamily	5.56E-03	331406
	ORF140	99.96	99.87	0.12	0.37	-	-	-	-
	ORF207	99.96	99.87	0.07	0.20	putative Gp5 baseplate hub subunit and tail lysozyme	NLPC_P60 superfamily	3.15E-35	328779
	ORF59	99.97	99.87	0.08	0.38	-	-	-	-
	ORF72	99.91	99.87	0.08	0.18	-	-	-	-

Divergent Core

ORF188	99.96	99.86	0.13	0.40	-	-	-	-
ORF124	99.63	99.86	0.46	0.16	-	-	-	-
ORF204	99.93	99.85	0.06	0.14	ribonucleoside diphosphate reductase, beta chain	Ferritin_like superfamily	1.00E-52	320867
ORF58	99.89	99.85	0.09	0.12	DNA polymerase	DNA_pol_A superfamily	5.25E-33	322025
ORF169	99.95	99.85	0.10	0.30	-	-	-	-
ORF202	99.95	99.84	0.15	0.46	-	-	-	-
ORF144	99.95	99.84	0.16	0.48	-	-	-	-
ORF36	99.85	99.83	0.14	0.23	-	-	-	-
ORF11	99.95	99.83	0.16	0.49	-	-	-	-
ORF215	99.90	99.83	0.08	0.14	DNA ligase	CDC9 superfamily	1.57E-99	330238
ORF28	99.88	99.82	0.23	0.37	-	HNHc	1.08E-05	238038
ORF122	99.86	99.82	0.12	0.22	putative major head protein	Phage_cap_E superfamily	6.03E-24	309113
ORF155	99.87	99.81	0.37	0.56	-	-	-	-
ORF78	99.88	99.81	0.13	0.26	-	-	-	-
ORF151	99.93	99.80	0.19	0.59	-	-	-	-
ORF208	99.88	99.80	0.12	0.22	PhoH family protein	P-loop_NTPase superfamily	4.93E-31	328724
ORF53	99.89	99.78	0.16	0.31	-	-	-	-
ORF172	99.91	99.77	0.14	0.33	-	-	-	-
ORF25	99.92	99.76	0.23	0.70	-	-	-	-
ORF192	99.66	99.74	0.45	0.49	-	-	-	-
ORF145	99.70	99.74	0.46	0.53	-	-	-	-
ORF27	99.91	99.73	0.18	0.55	-	-	-	-
ORF8	99.91	99.72	0.15	0.44	-	-	-	-
ORF6	99.86	99.72	0.31	0.83	-	-	-	-
ORF130	99.83	99.71	0.12	0.26	-	-	-	-
ORF127	98.84	99.71	2.24	0.47	-	-	-	-
ORF193	99.83	99.70	0.13	0.25	putative thymidylate synthase	Thy1 superfamily	1.56E-04	332234
ORF152	99.90	99.69	0.10	0.30	-	-	-	-
ORF220	99.89	99.67	0.17	0.52	-	-	-	-
ORF121	99.90	99.67	0.07	0.21	-	T5orf172	8.07E-05	313714
ORF143	99.85	99.67	0.26	0.53	-	-	-	-
ORF133	99.77	99.66	0.18	0.40	-	-	-	-
ORF128	99.88	99.65	0.11	0.34	terminase large subunit	Terminase_6 superfamily	7.55E-28	321850
ORF40	99.85	99.64	0.14	0.34	-	-	-	-
ORF217	99.88	99.64	0.19	0.58	-	-	-	-
ORF184	99.88	99.63	0.16	0.50	-	-	-	-
ORF187	99.84	99.63	0.23	0.50	-	-	-	-
ORF150	99.88	99.63	0.20	0.60	-	-	-	-
ORF70	99.82	99.59	0.09	0.30	-	-	-	-
ORF31	99.86	99.59	0.17	0.51	-	-	-	-
ORF185	99.75	99.58	0.21	0.40	-	-	-	-
ORF51	99.85	99.55	0.17	0.52	-	-	-	-
ORF212	99.90	99.55	0.19	0.72	-	-	-	-
ORF77	99.70	99.55	0.63	0.64	-	-	-	-
ORF156	99.85	99.55	0.24	0.72	-	-	-	-
ORF136	99.85	99.53	0.25	0.75	-	-	-	-
ORF200	99.84	99.52	0.19	0.58	-	-	-	-
ORF186	99.84	99.51	0.22	0.66	-	-	-	-
ORF209	99.84	99.50	0.14	0.43	-	-	-	-
ORF123	99.81	99.48	0.24	0.57	-	-	-	-
ORF108	99.83	99.48	0.27	0.83	-	-	-	-
ORF107	99.04	99.48	1.41	0.64	-	-	-	-
ORF199	99.82	99.44	0.22	0.67	-	-	-	-
ORF203	99.63	99.42	0.83	1.27	-	Ribonuc_red_lgC superfamily	3.16E-87	332162
ORF125	99.13	99.42	0.94	0.46	-	Peptidase_S78_2 superfamily	4.65E-10	317012
ORF205	99.81	99.42	0.19	0.56	-	-	-	-
ORF219	99.72	99.41	0.26	0.70	-	-	-	-
ORF37	99.74	99.19	0.26	0.78	-	-	-	-
ORF165	99.30	99.18	1.32	1.43	-	-	-	-
ORF129	99.62	99.15	0.51	1.36	-	-	-	-
ORF141	99.72	99.14	0.27	0.82	-	-	-	-
ORF198	99.62	99.10	0.29	0.63	-	-	-	-
ORF201	99.70	99.09	0.30	0.93	-	-	-	-
ORF67	99.70	99.08	0.32	0.97	-	-	-	-
ORF110	99.63	99.00	0.63	1.60	-	-	-	-
ORF69	99.67	98.99	0.22	0.68	-	-	-	-
ORF94	99.20	98.90	1.98	2.19	-	-	-	-
ORF103	96.62	98.87	3.49	0.87	-	-	-	-
ORF93	97.56	98.77	3.12	1.64	-	-	-	-
ORF84	97.62	98.67	3.12	1.99	-	DUF3383 superfamily	4.95E-09	314693
ORF189.1	99.52	98.54	0.58	1.76	-	-	-	-
ORF197	99.46	98.34	0.42	1.30	-	-	-	-
ORF157	99.15	98.03	0.94	1.79	-	-	-	-
ORF113	97.85	97.96	2.06	2.60	-	NRDD superfamily	5.49E-149	330954
ORF174	97.92	97.76	8.27	8.40	-	T5orf172	1.47E-04	313714
ORF91	97.11	97.59	2.66	2.23	-	-	-	-
ORF92	96.81	97.49	3.76	2.96	-	-	-	-
ORF161	94.88	97.41	6.15	3.11	-	-	-	-
ORF35	99.45	97.16	1.10	5.70	-	-	-	-
ORF87	94.86	97.13	4.65	2.77	-	-	-	-
ORF119	97.97	97.12	2.17	2.95	-	-	-	-
ORF86	94.49	97.05	5.22	2.98	-	-	-	-
ORF85	94.13	96.50	7.18	4.47	-	BF2867_like_C	2.18E-03	240526
ORF159	96.66	96.04	3.20	3.89	-	-	-	-
ORF104	96.45	96.00	5.71	6.21	-	-	-	-
ORF221	95.99	95.40	5.24	5.88	-	-	-	-
ORF97	95.94	95.27	6.13	6.91	-	PP-binding superfamily	1.14E-07	324546
ORF5	99.32	92.28	0.85	10.28	-	GIY-YIG_SF superfamily	5.49E-03	326551
ORF114	91.57	91.83	6.46	6.32	-	Radical_SAM superfamily	1.93E-74	327492

Table S1: Core-genome information. The data for each ORF represented in Figure 4 is listed in columns 2 and 3. Columns 4 and 5 contain the standard deviations for those pairwise similarity values. The other columns contain information about putative gene function and conserved domain homology.

# Occurances	ORF Name	ICP1	ICP1_2001_A	ICP1_2001_B	ICP1_2001_C	ICP1_2001_D	ICP1_2001_E	ICP1_2001_F	ICP1_2004_A	ICP1_2005_A	ICP1_2006_A	ICP1_2006_B	ICP1_2006_C	ICP1_2006_D	ICP1_2006_E	ICP1_2009_A	ICP1_2011_A	ICP1_2011_B	ICP1_2011_C	ICP1_2012_A	Putative Function	Domain Name	E-Value	PSSM_ID
XVIII	ORF19	■	■	■	■	■	■	■	■	■	■	■	■	■	■	■	■	■	■		-	-	-	-
	ORF20	■	■	■	■	■	■	■	■	■	■	■	■	■	■	■	■	■	■		-	-	-	-
	ORF38	■	■	■	■	■	■	■	■	■	■	■	■	■	■	■	■	■	■		-	-	-	-
	ORF95	■	■	■	■	■	■		■	■	■	■	■	■	■	■	■	■	■	■	-	-	-	-
	ORF96	■	■	■	■	■	■		■	■	■	■	■	■	■	■	■	■	■	■	-	-	-	-
	ORF105	■	■	■	■	■	■	■	■	■	■	■	■	■	■		■	■	■	■	-	-	-	-
	ORF106	■	■	■	■	■	■	■	■	■	■	■	■	■	■		■	■	■	■	-	-	-	-
	ORF111	■	■	■	■	■	■		■	■	■	■	■	■	■	■	■	■	■	■	-	-	-	-
	ORF178	■	■	■	■	■	■		■	■	■	■	■	■	■	■	■	■	■	■	-	-	-	-
	ORF180.1	■	■	■	■	■	■	■	■	■	■	■	■	■	■	■	■	■	■		-	-	-	-
	ORF182	■	■	■	■	■	■		■	■	■	■	■	■	■	■	■	■	■	■	-	-	-	-
	ORF183	■	■	■	■	■	■	■		■	■	■	■	■	■	■	■	■	■	■	-	-	-	-
XVII	ORF146	■	■	■	■	■	■	■	■	■	■	■			■	■	■	■	■	■	-	-	-	-
	ORF148	■	■	■	■	■	■	■	■	■	■	■			■	■	■	■	■	■	-	-	-	-
	ORF147	■	■	■	■	■	■	■	■	■	■	■			■	■	■	■	■	■	helicase	P-loop_NTPase super family	5.71e-12	328724
	ORF149	■	■	■	■	■	■	■	■	■	■	■			■	■	■	■	■	■	-	-	-	-
XVI	ORF21	■	■	■	■	■	■	■	■	■	■	■	■	■	■	■	■				-	-	-	-
XV	ORF14	■	■	■	■	■	■	■	■	■	■	■	■	■	■					■	-	-	-	-
	ORF98	■	■	■	■	■	■	■	■	■	■	■	■	■	■					■	-	-	-	-
	ORF99	■	■	■	■	■	■	■	■	■	■	■	■	■	■					■	-	-	-	-
	ORF100	■	■	■	■	■	■	■	■	■	■	■	■	■	■					■	-	-	-	-
	ORF101	■	■	■	■	■	■	■	■	■	■	■	■	■	■					■	-	-	-	-
	ORF222	■	■	■	■	■	■	■	■	■	■	■	■	■	■					■	-	-	-	-
	ORF223	■	■	■	■	■	■	■	■	■	■	■	■	■	■					■	-	-	-	-
	ORF225	■	■	■	■	■	■	■	■	■	■	■	■	■	■					■	-	-	-	-
XIV	ORF16	■	■	■	■	■	■	■	■						■	■	■	■	■	■	-	-	-	-
	ORF114.1		■	■		■	■		■	■	■	■			■	■	■	■	■	■	-	-	-	-
	ORF228	■	■	■	■	■		■		■	■	■				■	■	■	■	■	-	Pribosyltran_N superfamily	1.78166E-22	330902
	ORF229	■	■	■	■	■		■		■	■	■				■	■	■	■	■	-	PRK09198	0	236407
XIII	ORF89	■	■	■	■	■	■	■		■	■	■	■	■						■	antirepressor protein	Bro-N superfamily	3.42643E-23	324611
	ORF90	■	■	■	■	■	■	■		■	■	■	■	■						■	-	-	-	-
	ORF102	■	■	■	■	■	■	■		■	■	■	■	■	■						-	-	-	-
	ORF114.2		■	■		■	■			■	■	■			■	■	■	■	■	■	-	-	-	-
XII	ORF88	■	■	■	■	■	■	■			■	■	■	■						■	-	T5orf172	2.97023E-06	313714
XI	ORF22	■	■		■					■	■	■	■	■		■	■			■	-	-	-	-
X	ORF24	■	■		■					■	■	■	■	■		■	■				-	-	-	-
IX	ORF15.1									■	■	■	■	■		■	■	■	■		-	-	-	-
	ORF17	■	■	■	■	■	■	■	■						■						-	-	-	-
	ORF18	■	■	■	■	■	■	■	■						■						-	-	-	-
	Cas1						■	■	■	■					■	■	■	■	■		Cas1	Cas1_I-II-III superfamily	8.59874E-89	321096
	Cas3						■	■	■	■					■	■	■	■	■		Cas3	P-loop_NTPase superfamily	3.8104E-118	328724
	Csy1						■	■	■	■					■	■	■	■	■		Csy1	Csy1_I-F superfamily	4.37493E-05	324501
	Csy2						■	■	■	■					■	■	■	■	■		Csy2	Csy2_I-F superfamily	1.24047E-14	325199
	Csy3						■	■	■	■					■	■	■	■	■		Csy3	Csy3_I-F superfamily	1.6744E-104	324502
	Csy4						■	■	■	■					■	■	■	■	■		Csy4	Cas6_I-F superfamily	2.50407E-28	324504
VII	ORF23	■	■							■	■	■	■	■							-	-	-	-
	ORF87.1						■		■						■	■	■	■	■		-	-	-	-
	putative_Rha_protein						■		■						■	■	■	■	■		Rha_protein	Phage_pRha	5.83871E-25	312982
V	ORF115	■			■			■					■	■							-	-	-	-
	ORF116	■			■			■					■	■							HNH endonuclease	-	-	-
	ORF117	■			■			■					■	■							-	-	-	-
	ORF117.1	■			■			■					■	■							-	-	-	-
	ORF118	■			■			■					■	■							-	-	-	-
	ORF160	■	■	■	■	■															-	-	-	-
	ORF162	■	■	■	■	■															-	-	-	-
	ORF163	■	■	■	■	■															-	-	-	-
	ORF113x		■	■		■	■								■						-	NRDD superfamily	8.63697E-73	330954
	putative_DNA-methyltransferase						■		■				■	■	■						DNA-methyltransferase	AdoMet_MTases superfamily	2.75054E-52	327401
IV	ORF97.1															■	■	■	■		-	-	-	-
	ORF181.1						■		■				■	■							-	-	-	-
	ORF5x															■	■	■	■		-	-	-	-
	HigA															■	■	■	■		-	antidote_HigA	4.07815E-27	274228
II	ORF15.2												■	■							-	-	-	-
	ORF15.3												■	■							-	PTH2_family superfamily	0.000800162	327521
	ORF145.1												■	■							-	-	-	-
	ORF147.1												■	■							-	-	-	-
	ORF165x		■																	■	-	-	-	-
	ORF174x												■	■							-	T5orf172	0.000295242	313714
	ORF192x			■		■															-	-	-	-
	ORF87p						■	■													-	-	-	-
I	ORF22.1																			■	-	-	-	-
	ORF94.1							■													-	-	-	-
	ORF94.2							■													-	-	-	-
	ORF96.1							■													-	PP-binding superfamily	1.18907E-09	324546
	ORF104.1															■					-	-	-	-
	ORF104.2															■					-	-	-	-
	ORF104.3															■					-	Lipase_GDSL_2	6.70426E-18	316033
	ORF104.4															■					-	-	-	-
	ORF179.1							■													-	-	-	-
	ORF228.1							■													-	-	-	-
	ORF203x																■				-	Ribonuc_red_lgC superfamily	5.56357E-84	332162
	ORF228x				■																-	PRTases_typeI superfamily	1.87368E-08	320892
	ORF86p							■													-	-	-	-
	putative_DNA-methyltransferase_x														■						DNA-methyltransferase_x	-	-	-
		ICP1	ICP1_2001_A	ICP1_2001_B	ICP1_2001_C	ICP1_2001_D	ICP1_2001_E	ICP1_2001_F	ICP1_2004_A	ICP1_2005_A	ICP1_2006_A	ICP1_2006_B	ICP1_2006_C	ICP1_2006_D	ICP1_2006_E	ICP1_2009_A	ICP1_2011_A	ICP1_2011_B	ICP1_2011_C	ICP1_2012_A				

Table S2: Accessory-genome ORF occurrence matrix. Each ORF in the accessory genome is listed along with any putative function or conserved domain homology information. A black square indicates that the ORF occurs in a specific genome.
